# Prevalence of thyroid dysfunction in older Chinese patients with type 2 diabetes—A multicenter cross-sectional observational study across China

**DOI:** 10.1371/journal.pone.0216151

**Published:** 2019-05-02

**Authors:** Yu Zhu, Fengmei Xu, Jie Shen, Youshuo Liu, Changhua Bi, Jing Liu, Yufeng Li, Xueqin Wang, Zhengnan Gao, Linlang Liang, Yanyan Chen, Weiping Sun, Qingbo Guan, Junqing Zhang, Zuojie Luo, Lixin Guo, Xiaopin Cai, Ling Li, Lingling Xiu, Li Yan, Chunlin Li, Xiaoyun Shi, Mei Zhu, Jian Kuang, Guangwei Li, Linong Ji

**Affiliations:** 1 Peking University People’s Hospital, Beijing, China; 2 General Hospital of Hebi Coal Industry [Group] Co., Ltd., Hebi, China; 3 The Third Affiliated Hospital of Southern Medical University, Guangzhou, China; 4 The Second Xiangya Hospital of Central South University, Changsha, China; 5 CNPC Central Hospital, Hebei, China; 6 Gansu Provincial Hospital, Lanzhou, China; 7 Beijing Pinggu Hospital, Beijing, China; 8 Beijing Huairou Hospital of University of Chinese Academy Sciences, Beijing, China; 9 Dalian Municipal Central Hospital Affiliated of Dalian Medical University, Dalian, China; 10 The General Hospital of Shenyang Military Region, Shenyang, China; 11 Fuwai Hospital Chinese Academy of Medical Sciences, Beijing, China; 12 The First People’s Hospital of Xiangtan City, Xiangtan, China; 13 Shandong Provincial Hospital, Shandong, China; 14 Peking University First Hospital, Beijing, China; 15 The First Affiliated Hospital of Guangxi Medical University, Guangxi, China; 16 Beijing Hospital, Beijing, China; 17 China-Japan Friendship Hospital, Beijing, China; 18 Shengjing Hospital of China Medical University, Shenyang, China; 19 First Affiliated Hospital of Sun Yat-sen University, Guangzhou, China; 20 Sun Yat-sen Memorial Hospital of Sun Yat-sen University, Guangzhou, China; 21 The General Hospital of the People's Liberation Army [301 Hospital], Beijing, China; 22 The General Hospital of Chinese People's Armed Police Forces, Beijing, China; 23 General Hospital, Tianjin Medical University, Tianjin, China; 24 Guangdong General Hospital, Guangzhou, China; Universidad Miguel Hernandez de Elche, SPAIN

## Abstract

Type 2 diabetes [T2D] and thyroid dysfunction [TD] often co-occur, have overlapping pathologies, and their risk increases with age. Since 1995, universal salt iodization has been implemented in China to prevent disorders caused by iodine deficiency. However, after two decades of implementation of universal salt iodization, the prevalence of TD in elderly Chinese patients with T2D is not well described and may have been underestimated. We conducted a questionnaire-based survey across 24 endocrinology centers in China between December 2015 and July 2016. Demographic and clinical data from 1677 patients with T2D were obtained and analyzed to examine the prevalence of TD along with T2D in these patients. We assessed TD prevalence according to the four TD subtypes [subclinical hypothyroidism, clinical hypothyroidism, subclinical hyperthyroidism, and clinical hyperthyroidism], TD history, gender, and age. The diagnosis rates were calculated for TD and also for the TD subtype. The number of patients reaching treatment goals for T2D [hemoglobin A1c <7%] and TD [normal free thyroxine and thyroid-stimulating hormone [TSH]] and the incidences of complications and comorbidities were recorded. Among the enrolled patients with T2D [N = 1677], TD was diagnosed in 23.79% [399/1677] out of which 61% (245/399) were previously diagnosed and 38.59% (154/399) were newly diagnosed cases. Subclinical hypothyroidism, clinical hypothyroidism, subclinical hyperthyroidism, and clinical hyperthyroidism were reported in 4.89%, 9.3%, 1.13%, and 3.16% of the total population, respectively. Among patients previously diagnosed with TD, the incidence in women [166/795; 20.88%] was higher than in men [79/882; 8.96%]. The treatment goals for TD and T2D were attained in 39.6% [97/245] and 34.41% [577/1677] of the cases, respectively. Diabetic complications and comorbidities were reported in 99.7% of patients, with peripheral neuropathy being the most common [43.46%] followed by cataract [24.73%]. We had found that the incidences of dyslipidemia, elevated LDL levels, and osteoporosis were significantly higher in patients with TD than those without TD. TD is underdiagnosed in elderly Chinese patients with T2D.

## Introduction

Worldwide, the proportion of elderly population is increasing. The global proportion of people aged ≥60 years increased from 9.2% in 1990 to 11.7% in 2013 and is expected to reach 21.1% by 2050 [1]. China is no exception to this; as of 2015, 16.1% [>220 million people] of the total population in China is aged >60 years [2]. There are several diseases particularly targeting the elderly population, and diabetes mellitus is one of them [3].

In 2013, it was estimated that 10.9% of the Chinese population had either diagnosed or undiagnosed diabetes, whereas 35.7% of them were in prediabetic stage [4] with a higher prevalence in the elderly population [4]. Thyroid dysfunction [TD] is another disease that is known to be prevalent in the elderly population [5]. Since 1995, universal salt iodization has been implemented in China to prevent iodine deficiency diseases. However, in a 5 years follow-up study, prevalence of hypothyroidism and autoimmune thyroiditis increased with adequate or excessive iodine intake [6]. Following universal salt iodization, subclinical hypothyroidism is reported to be the most commonly observed TD subtype in China [6]. A meta-analysis of data from Chinese population [N = 178,995] reported association of different levels of iodine intake with thyroid disease [7]. In this study, subjects were classified into 3 subgroups based on the median urinary iodine concentrations: low-iodine group [<100mg/L]; medium-iodine group [100 to 299mg/L]; high iodine group [>300mg/L] [7]. The prevalence of clinical hyperthyroidism and subclinical hyperthyroidism was 0.7%. and 1.2%, respectively. There was no significant difference in prevalence of hyperthyroidism in each group. The least prevalence observed was of hypothyroidism [0.2%] in the medium-iodine group, whereas the highest was for subclinical hypothyroidism [8.3%] in the high iodine group. However, subgroup analysis according to the age was not performed, and so, prevalence in elderly population was not reported.

Diabetes mellitus and TD are two endocrine-related disorders most commonly encountered in clinical practice [8], and their association is marked by a complex interdependence [9]. They share several pathologies and are known to co-occur frequently [10,11]: approximately 12%–15% of patients with type 2 diabetes [T2D] were reported to have TD [9,12,13] as compared with 1.3%–4.6% of the general population [14,15]. In addition, both hyper- and hypothyroidism are associated with insulin resistance in patients with T2D [10]. Hyperthyroidism promotes hyperglycemia [16] and reduces the half-life of insulin [17,18], and hence, the dosage of insulin needed to achieve normal blood glucose levels is likely to be higher in T2D patients with hyperthyroidism [19]; conversely, patients with T2D suffering from hypothyroidism may require less insulin [10]. With the reports of subclinical hypothyroidism being independently associated with severe diabetic retinopathy, undiagnosed TD in patients with diabetes can adversely affect their metabolic profiles and thereby increase the risk of diabetic complications [20,21]. Therefore, it is imperative to diagnose TD in patients with T2D as early as possible to plan an effective treatment because in most of the cases, TD is asymptomatic [9].

In spite of evidence of a high prevalence of TD among patients with T2D globally, there is limited information on the prevalence of TD in elderly Chinese patients with T2D. Moreover, the profile of TD prevalence among Chinese elderly patients based on clinical and demographic parameters is unknown, which might be helpful in introducing relevant guidelines. Therefore, this cross-sectional study evaluated the prevalence of TD among elderly patients with T2D who visited endocrinology clinics in China [CROSS-DT Study]. In addition, we determined the profile of TD among enrolled patients according to the TD subtype, history of TD, gender, age, attainment of treatment goals, complications and comorbidities, and diagnosis rates.

## Materials and methods

### Study design

This was an observational cross-sectional study carried out by the Chinese Association of Geriatric Research [CAGR] as a questionnaire-based survey in 24 outpatient endocrinology clinics in China between December 2015 and July 2016. The 24 study sites selected were members of CAGR and were willing to participate in the study. The study sites were spread over the first, second, third, fourth, and fifth line cities in all geographical regions of China, had tier 1, 2, and 3 hospitals, and represented different levels of economic development, ensuring that the results of the study were not biased. The leading study site was Peking University People’s Hospital with Dr Linong Ji being the principal investigator.

### Ethics statement

This study was performed in accordance with the tenets of the Declaration of Helsinki and approved by the ethical committee of Peking University People’s Hospital and written informed consent was obtained from all participants.

#### Study participants

Patients aged ≥60 years with a confirmed diagnosis of T2D as per the criteria of World Health Organization, 1999 [22], were included in the study. Patients diagnosed with type 1 diabetes and those not willing to participate in the study were excluded from the study. Only patients who provided written informed consent were included in the study.

#### Data collection

Data were collected using case report forms, which the sub-investigators at each clinic completed by consulting the patients and returned to the principal site. Demographic details including age, gender, race, duration for which the patients were affected by diabetes, education, history of smoking or alcoholism, and physical activities were obtained. All eligible patients also underwent a general physical examination and a medical chart review at the time of recruitment into the study.

A detailed medical history of diabetes and its complications [diabetic nephropathy, retinopathy, neuropathy, foot, and frequency of hypoglycemia], TD, hypertension, dyslipidemia, coronary heart disease, cerebrovascular disease, and other diseases was recorded.

Diabetic complications were diagnosed based on the medical records. Presence of glaucoma, cataract, retinopathy and blindness were considered as diabetic ophthalmopathy while diabetic nephropathy was diagnosed according to urinary albumin to creatinine ratio (>30mg/g) and/or eGFR (< 60ml/min). Diabetic peripheral neuropathy was diagnosed based on clinical symptoms of neuropathy (pain, numbness and abnormal sensation) and any one abnormality in the tests for ankle reflex, acupuncture pain sensation, vibration sensation, pressure sensation and temperature sensation. Patients without clinical symptoms of neuropathy, but with more than one abnormality in the above-mentioned tests were also considered to be diabetic peripheral neuropathy. Diabetic foot was diagnosed by foot ulcer with infection and/or amputation. Lower extremity atherosclerotic disease was diagnosed by color ultrasound examination.

The specific treatment details regarding previous [last 6 months] and concomitant medications were also obtained. Thyroid test results [TSH, free triiodothyronine [FT3], free thyroxine [FT4], total T3, and total T4, of which TSH, FT3, and FT4 were mandatory] and other laboratory test results in the previous year were recorded, and thyroid tests were conducted for those who did not undergo thyroid tests within the past one year. Moreover, the past one year [if available] data on hemoglobin A1c [HbA1c], total cholesterol, low density lipoproteins [LDLs], triglycerides, electrocardiograms, and bone density were collected. Missing data for demographic and laboratory parameters were recorded in the case report form.

The diagnosis of TD was made according to textbook definitions and guidelines [23,24]. Because each hospital site used different laboratory kits for hormone measurement, there was no consistent cutoff value. Rather, each hospital performed a diagnosis according to its own normal range values. However, in general, the following definitions were used:

- Clinical hyperthyroidism [any one of the below criteria]:
  ○ decreased TSH with elevated FT4 and /or elevated FT3
  ○ subjects with a history of hyperthyroidism who were receiving anti-thyroid agents

- Clinical hypothyroidism: decreased FT4 and elevated TSH [including patients with a history of hypothyroidism who were receiving levothyroxine]
- Subclinical hyperthyroidism: normal FT4, normal FT3, and decreased TSH
- Subclinical hypothyroidism: normal FT4 and elevated TSH

Based on these definitions, the investigator diagnosed TD if an abnormal TSH or FT4 value was found, even in patients without any history of thyroid disease.

The medical records were re-analyzed by endocrinologists for previously diagnosed thyroid dysfunction.

#### Study end points

The primary end point of this study was the prevalence of TD in elderly Chinese patients with T2D. The secondary end points were ascertaining the prevalence rates by TD subtype, gender, age, and history of TD, the percentage of patients reaching treatment goals for both T2D and TD, and the percentage of complications and comorbidities in elderly patients with T2D. The diagnosis rates [proportion of patients with T2D and with previously diagnosed TD] were calculated for each TD subtype.

### Statistical analysis

In order to detect an expected prevalence rate of 15%, with 2% precision and 95% confidence, 1650 subjects were required. The sample size was calculated as follows:

$$n=Z^{2}p[1‐p]/d^{2}$$

[where n = sample size, Z = Z statistic for level of confidence, p = expected prevalence or proportion, and d = precision].

For continuous variables [age, height, and body mass index], the results were summarized using the number of observations [N], number of missing data points [miss], means, standard deviations [SDs], medians, 25th percentiles [Q1], 75th percentiles [Q3], minimums, and maximums. For categorical variables [gender and hypertension], the results were summarized as frequencies and percentages. Student’s t-test and chi-square test/Fisher’s exact test were used to determine the homogeneity of baseline characteristics. All statistical tests were two-sided and p < .05 was considered significant.

The statistical analysis was carried out using statistical analysis system [SAS] version 9.4 [SAS Institute Inc., Cary, North Carolina, USA].

## Results

### Study population

**Fig 1:**
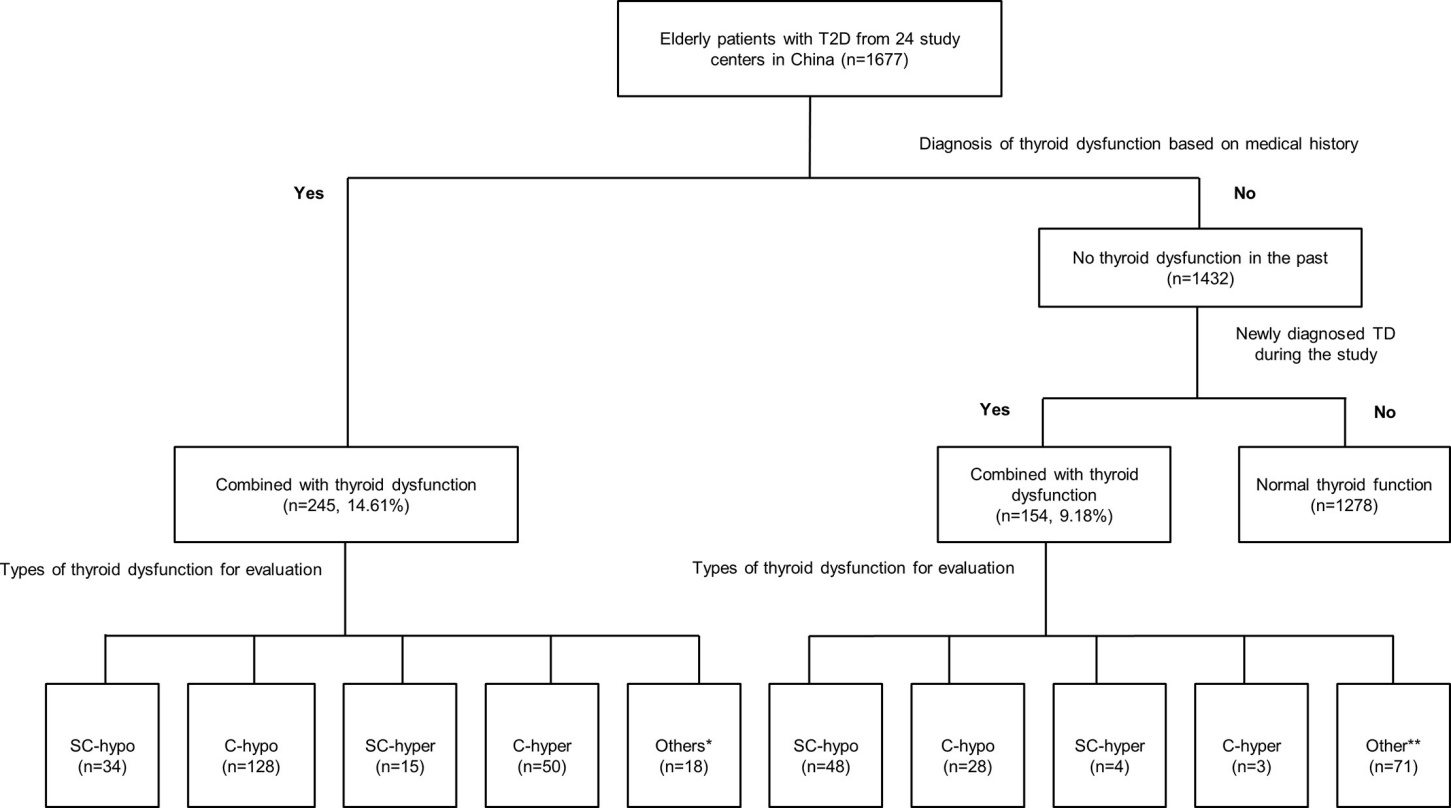
Flow chart showing the distribution of the population with thyroid dysfunction [TD] among elderly Chinese patients with type 2 diabetes [T2D]. Patients with a past medical history of thyroid dysfunction [TD]. **Other types of TD such as low T3. C: clinical; SC: subclinical; hypo: hypothyroidism; hyper: hyperthyroidism.

We enrolled 1677 patients [mean ± SD age: 71.17 ± 8.06 years; 882 [52.59%] male and 795 [47.41%] female patients] from 24 endocrinology centers across China. The demographic and baseline characteristics of the patients are summarized in Table 1. Fig 1, describes the patient enrolment according to eligibility criteria.

**Table 1:** Demographic and baseline characteristics of elderly, T2D patients with and without previously diagnosed TD [N = 1677].

Parameters mean [SD] or N [%]	All patients [N = 1677]	Without TD [n = 1432]	With previously diagnosed TD [according to medical history] [n = 245]
Age [years; mean [SD]]	71.17 [8.06]	71.26 [8.19]	70.61 [7.28]
<65 years	459 [27.37]	390 [27.23]	69 [28.16]
65≤ to <70 years	396 [23.61]	332 [23.18]	64 [26.12]
70≤ to <75 years	305 [18.19]	262 [18.30]	43 [17.55]
≥75 years	517 [30.83]	448 [31.28]	69 [28.16]
Gender, n [%]			
Male	882 [52.59]	803 [56.08]	79 [32.24]
Female	795 [47.41]	629 [43.92]	166 [67.76]
Marital status, n [%]			
Unmarried	5 [0.30]	4 [0.28]	1 [0.41]
Married	1578 [94.10]	1356[94.69]	222 [90.61]
Divorced	12 [0.72]	10 [0.70]	2 [0.82]
Widowed	73 [4.35]	55 [3.84]	18 [7.35]
Education, n [%]			
Illiterate	47 [2.80]	40 [2.79]	7 [2.85]
Primary school	381 [22.72]	336 [23.46]	45 [18.37]
Middle school	608 [36.25]	503 [35.13]	105 [42.86]
College degree or above	598 [35.66]	516 [36.03]	82 [33.47]
Height [cm; mean [SD]]	163.87 [8.20]	164.15 [8.27]	162.19 [7.62]
Weight [kg; mean [SD]]	66.92 [11.50]	67.14[11.54]	65.61 [11.18]
BMI [mean [SD]]	24.84 [3.37]	24.84 [3.38]	24.87 [3.35]
HR [beats per minute; mean [SD]]	75.49 [9.74]	75.53 [9.67]	75.22 [10.12]
SBP [mm Hg; mean [SD]]	134.08 [16.76]	134.55 [16.91]	131.32 [15.59]
DBP [mm Hg; mean [SD]]	76.93 [9.47]	77.21 [9.45]	75.29 [9.44]

BMI: body mass index; DBP: diastolic blood pressure; HR: heart rate; SBP: systolic blood pressure; SD: standard deviation; T2D: type 2 diabetes; TD: thyroid dysfunction.

The patients’ medical histories and clinical data at baseline are summarized in Table 2. All included patients had thyroid function test results [either from their medical history or from the new test] for the 12 months prior to inclusion in the study. The mean duration of T2D was 12.21 ± 7.56 years and 3.19 ± 2.80 months in patients with a disease course of >1 year and <1 year, respectively, with corresponding durations of TD of 9.28 ± 9.81 years and 2.93 ± 2.75 months, respectively.

**Table 2:** Baseline medical history and course of disease in elderly, T2D patients [N = 1677].

History	n [%]
Course of T2D	
T2D ≥1 year	1524 [90.88]
T2D <1 year	129 [7.69]
Uncertain	16 [0.95]
Missing	8 [0.48]
TD [last 12 months]	245 [14.61]
Type of previously diagnosed TD	
Hyperthyroidism	50 [2.98]
Hypothyroidism	128 [7.63]
SC hyperthyroidism	15 [0.89]
SC hypothyroidism	34 [2.03]
Uncertain	18 [1.07]
Hypertension* [n = 1664]	1070 [64.30]
Dyslipidemia* [n = 1661]	725 [43.65]
Type of dyslipidemia*^,^^†^ [n = 641]	
TC abnormal	287 [44.77]
LDL abnormal	281 [43.84]
HDL abnormal	127 [19.81]
TG abnormal	254 [39.63]
CHD* [n = 1666]	497 [29.83]
CVD* [n = 1670]	324 [19.40]
Osteoporosis* [n = 1661]	200 [12.04]
Pituitary disease* [n = 1669]	7 [0.42]

*Percentages based on the number of patients for whom data were available; the total number of patients with available data is shown in brackets for each comorbidity.

† Note that some patients had more than one type of dyslipidemia, and hence, the total number of dyslipidemia incidence is higher than the number of patients with dyslipidemia.

CHD: coronary heart disease; CVD: cerebrovascular disease; HDL: high- density lipoprotein; LDL: low- density lipoprotein; SC: subclinical; T2D: type 2 diabetes; TC: total cholesterol; TG: triglyceride.

Family histories of diabetes, thyroid disease, and coronary heart disease were reported in 471 [28.15%], 40 [2.40%], and 230 [14.08%] patients, respectively. Compared with T2D patients without TD, higher number of T2D patients with previously diagnosed TD had a family history of TD [1.61% vs. 6.97%; p < .0001].

The data on concomitant medications showed that 36 [2.15%] patients were receiving anti-thyroid agents, whereas 113 [6.74%] were receiving levothyroxine. The most common medication taken by the enrolled patients were antihypertensive agents [59.15%], followed by lipid-lowering agents [56.11%], antiplatelet aggregating agents [53.22%], and others [29.99%]. The number of concomitant medications used by patients with and without TD was similar.

### Prevalence TD in patients with T2D: overall and by age, gender, TD subtype and history of TD

The overall prevalence of TD in the elderly patients with T2D enrolled for this study was 23.79% [399 of 1677 patients].

A total of 14.61% [245/1677] of enrolled patients had a previously diagnosed TD [of which, 67.76% were female patients], whereas 9.18% were newly diagnosed [154/1677] with TD. Fig 1 shows the distribution of the population affected by TD among elderly Chinese patients with T2D according to TD history and subtype. Of the 399 patients with TD, the diagnosis rate [that is, the proportion of patients with previously diagnosed TD] was 61.4% [245/399], that is, 38.6% [154/399] of TD cases were previously undiagnosed. Clinical hypothyroidism was the major disease subtype in patients with previously diagnosed TD, [7.63%; 128/1677]. In contrast, subclinical hypothyroidism was the major disease subtype in newly diagnosed patients [2.98%; 50/1677].

In the previously diagnosed TD, the prevalence was higher in female [20.88%] than in male [8.96%] subjects [p < .0001]. However, age did not significantly affect the prevalence of TD. Proportion of patients with subclinical hypothyroidism, clinical hypothyroidism, subclinical hyperthyroidism, and clinical hyperthyroidism was 4.89% [82/1677], 9.30% [156/1677], 1.13% [19/1677], and 3.16% [53/1677], respectively. Further 5.31% [89/1677] of patients had “other” type of TD [example, low T3].

### Achievement of treatment goals

The treatment goals for previously diagnosed TD [normal FT4 and TSH] and T2D [HbA1c <7%] were achieved in 39.6% [97/245] and 34.41% [577/1677] of the cases, respectively. For calculation of the percentage of patients with TD who achieved treatment goal, only data of patients with previously diagnosed was used because patients newly diagnosed with TD had not commenced TD treatment. Higher proportion of patients with T2D and TD attained the T2D treatment goal [51.02%; p <0.0001] than those without TD [31.56%].

### Complications and comorbidities

**Fig 2:**
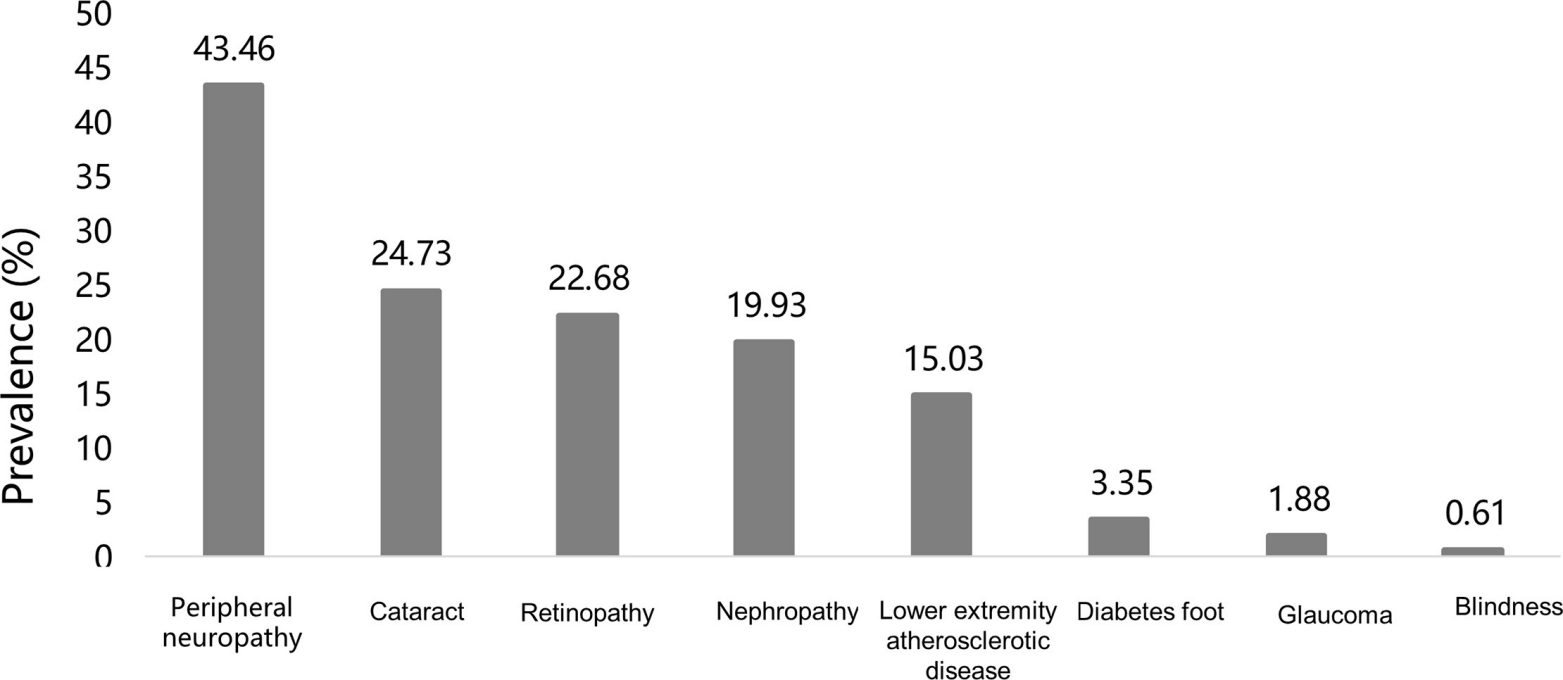
Prevalence of diabetic complications in elderly Chinese patients with type 2 diabetes.

Diabetic complications were reported in 99.7% patients [1672/1677; 880 males and 792 females] across all age groups with peripheral neuropathy [43.46%] being the most prevalent complication followed by cataract [24.73%], and diabetic retinopathy [22.68%] [Fig 2].

Incidences of concomitant dyslipidemia, elevated LDL levels, and osteoporosis were significantly higher in patients with previously diagnosed TD than those without TD [Table 3]. T2D patients with previously diagnosed TD had significantly higher incidence of diabetes [p = .0066], TD [p < .0001], and cardiovascular disease [p = .0016; Table 3] in family history than those without TD.

**Table 3:** Patient profiles, including comorbidities and family history, for elderly T2D patients suffering with and without previously diagnosed TD.

Comorbidity	With previously diagnosed TD	Without previously diagnosed TD	p-Value
Hypertension	160/245 [65.31]	910/1419 [64.13]	.9368
Dyslipidemia	135/243 [55.56]	590/1418 [41.61]	.0002
Type of dyslipidemia			
TC abnormal	64/127 [50.39]	223/514 [43.39]	.1549
LDL abnormal	67/127 [52.76]	214/514 [41.63]	.0237
HDL abnormal	23/127 [18.11]	104/514 [20.23]	.5909
TG abnormal	52/127 [40.94]	202/514 [39.30]	.7343
CHD	88/244 [36.07]	409/1422 [28.76]	.0598
CVD	47/245 [19.18]	277/1425 [19.44]	.9619
Osteoporosis	57/243 [23.46]	143/1418 [10.08]	< .0001
Pituitary disease	3/245 [1.22]	3/1424 [0.21]	.0444
**Family history of disease**			
Diabetes	87/245 [35.51]	384/1428 [26.89]	.0066
Thyroid disease	17/244 [6.97]	23/1426 [1.61]	< .0001
Cardiovascular disease	50/242 [20.66]	180/1391 [12.94]	.0016

Data are presented as n/N [%] where the N represents the number of patients for whom data were available for each comorbidity or family disease history.

CHD: coronary heart disease; CVD: cerebrovascular disease; HDL: high density lipoprotein; LDL: low density lipoprotein; TC: total cholesterol; TG: triglyceride.

### Diagnosis rate by TD subtype

The diagnosis rates by TD subtype [i.e., the proportion of patients with a particular subtype of TD who had a prior diagnosis] were 41.5%, 82.1%, 78.9%, and 94.3% for subclinical hypothyroidism, clinical hypothyroidism, subclinical hyperthyroidism, and clinical hyperthyroidism, respectively.

## Discussion

This study, evaluated the prevalence of TD in elderly patients with T2D in 24 endocrinology clinics across China and found that TD was underdiagnosed in this population. Our findings suggest that regular annual screening for thyroid function in patients with T2D maybe helpful to improve health outcomes and quality of life in elderly patients with T2D [13,25].

The overall prevalence of TD in this study was 23.79%, which was higher than the prevalence rates reported in some other studies on patients with T2D [12%–15%], [12,13] in the general [26,27] and healthy elderly [28,29] populations. Previous studies have reported highly varied prevalence rates of TD in patients with T2D [13,30–32]. An observational cross-sectional study conducted in India. reported a high TD prevalence of 25.31% in patients with T2D [13], which is similar to our findings, whereas other studies have reported lower prevalence estimates for TD in patients with T2D [12.5%–16%] [12,13,31]. The high prevalence of TD in elderly, T2D patients observed in our study indicates that we need focused TD screening for the comorbidities in this target population.

In the present study, the most common TD subtypes reported were clinical hypothyroidism [9.3%] and subclinical hypothyroidism [4.89%]; subclinical hypothyroidism was the most common subtype [31.2% [n = 48]] among 154 patients with newly diagnosed TD. Our results are supported by three other studies that reported subclinical hypothyroidism as the most common subtype of TD in patients with T2D: in a Spanish screening study, 64.5% patients with newly diagnosed TD had subclinical hypothyroidism [32]; in a cross-sectional study of patients with type 1 or 2 diabetes conducted in Brazil, 12% of patients with T2D had subclinical hypothyroidism [9]; annual screening of outpatients in Scotland revealed that 4.8% patients with T2D had subclinical hypothyroidism [33].

Consistent with the other studies [9,12,13], we observed that proportion of patients with previously diagnosed TD was higher [14.61%] than of those who were newly diagnosed with TD [9.18%]. Majority of the population with TD had previously diagnosed TD with a diagnosis rate [61.4%], which might be due to evident symptoms necessitating more frequent medical reviews with a physician. The lower diagnosis rate for subclinical hypothyroidism [41.5%] may be due to its asymptomatic nature and with nonspecific complaints making physicians unable to recognize subclinical hypothyroidism that consequently delayed TD screening. The incidence of dyslipidemia, elevated LDL levels, and osteoporosis were significantly higher in patients with TD than those without TD in our study population. Since complications were more common in T2D patients with TD than those without TD, screening for early diagnosis of TD is important in patients with T2D. The observed correlation between TD and concomitant disease needs further prospective intervention studies (intervention of thyroid dysfunction).

Although guidelines for screening vary among countries with different age cutoffs in different populations, they recommend active and regular screening for TD[34–37]. For example, the American Academy of Family Physicians recommends screening of asymptomatic adults aged ≥60 years for TD [37].

In our study, the most common complications and comorbidities in T2D were diabetic neuropathy [43.46%], cataracts [24.73%], and diabetic retinopathy [22.68%]. We used specific diagnostic criteria for each diabetic complication. The high prevalence of diabetic complications (99.7%) in the current study could be attributed to the advanced age of the study participants. The average age of the recruited patients was 71 years old, with 72% of them > 65 years old; more than 90% of the patients had diabetes for more than 12 years with 64.3% having hypertension and 43.6% having dyslipidemia. Hence, the prevalence of diabetic complications (microvascular and macrovascular complications) was so high. In past, the prevalence of diabetic complications has been assessed in several studies in patients with comorbid TD [20,38–40]. According to some studies, subclinical hypothyroidism is an independent risk factor for severe diabetic retinopathy in patients with T2D [20,21]. However, other studies report controversial risks of diabetic retinopathy in patients with TD and T2D [20,38].

Patients’ profiles, including comorbidities and family history, are also an important factor that clinicians consider while planning treatment of patients with T2D. In fact, the family history of TD is a risk factor for TD in patients with T2D; in a cross-sectional study carried out on 117 patients with both T2D and TD [mean age: 59.3 years; mean duration of T2D: 17.3 years] in Saudi Arabia, 84.2% and 14.7% patients had a family history of diabetes and TD, respectively [41]. Similarly, in our study, 35.51% of patients with both T2D and TD had a family history of diabetes and 6.97% had a family history of TD.

Several mechanisms underlie the relationship of TD and microvascular complications in T2D [10,42] with insulin resistance being observed in diabetes patients with both clinical and subclinical hyperthyroidism [43]. Potential pathological states that lead to microvascular complications in patients with T2D having comorbid TD include oxidative stress, dyslipidemia, and endothelial dysfunction [44,45].

A 2011 survey that examined the prevalence of TD among 15,008 subjects in 10 major cities of China. revealed that prevalence of C-hypothyroidism, SC- hypothyroidism, C-hyperthyroidism, and SC hyperthyroidism of 1.11%, 16.7%, 0.89%, and 0.72%, respectively [46]. The differences in reported prevalence between that study and the present study could be due to the differences in baseline characteristics of the study population as the mean age of population reported in that study was 45.5 years. Importantly, the authors confirmed a significant increase in the prevalence of all four TD subtypes from 1999 to 2011 [46].

One of the limitations of this study is its cross-sectional design. The sample was derived from an outpatient setting and may not be representative of the true population. Also, there is a possibility of selection bias because the patients were already under medical care. Although 24 endocrinology centers were in iodine sufficient areas, we did not determine urinary iodine concentrations. Further, due to limited resources, only one dosage lab examination could be done for TD which could be a potential limitation. In addition, our study did not evaluate the impact of diabetes-related risk factors on TD, though, a previous study revealed no significant relationships between and diabetes-related clinical parameters such as duration of diabetes, HbA1c levels and diabetic complications with TD [22].

Given the high prevalence rates of TD in patients with T2D, we recommend a systematic approach to thyroid testing in patients with T2D, as this may prove beneficial in the managing endocrine conditions in these patients. Future studies might be undertaken to determine the cost-effectiveness of thyroid function screening in patients with diabetes. In future, a stepwise objective protocol should be designed for the management of subclinical TD in patients with T2D. This may be helpful to prevent possible complications with comorbid conditions. Appropriately designed studies should also be conducted to study the association of TD with other factors in patients with T2D, for example, the duration and complications of diabetes. In addition, larger-scale epidemiological studies are needed to further investigate the prevalence of TD in Chinese patients with T2D.

## Conclusions

TD is prevalent in Chinese patients with T2D, with clinical- hypothyroidism being the most common TD subtype, which was more prevalent in women than in men. In addition, dyslipidemia, osteoporosis, and CHD are common complications in elderly Chinese patients with both T2D and TD. We recommend routine thyroid function testing of elderly patients with T2D in outpatient settings, especially of elderly females, and other old patients with risk factors, including those with a family history of diabetes, TD, and cardiovascular disease.

## Supporting information

S1 File STROBE_checklist_v4_combined_PlosMedicine.docx. (DOCX)
