# Supplementary material for: Prevalence of thyroid dysfunction in older Chinese patients with type 2 diabetes—A multicenter cross-sectional observational study across China
Source: PLoS One. 2019 May 2;14(5):e0216151. doi: 10.1371/journal.pone.0216151 (PMC6497275; doi:10.1371/journal.pone.0216151)
Supplement: S1 File — (DOCX) [file pone.0216151.s001.docx]

STROBE Statement—checklist of items that should be included in reports of observational studies

|  | Item No. | Recommendation | Page  No. | Relevant text from manuscript |
| --- | --- | --- | --- | --- |
| **Title and abstract** | 1 | (*a*) Indicate the study’s design with a commonly used term in the title or the abstract | 1& 3 | Multicenter cross-sectional observational study |
|  |  | (*b*) Provide in the abstract an informative and balanced summary of what was done and what was found | 3& 4 | A questionnaire-based survey across 24 endocrinology centers in China between December 2015 and July 2016. Thyroid dysfunction is underdiagnosed in elderly Chinese patients with Type-2 diabetes, which may affect their health outcomes. |
| Introduction | | | |  |
| Background/rationale | 2 | Explain the scientific background and rationale for the investigation being reported | 5-7 | Worldwide, the proportion of elderly population is increasing. There are several diseases particularly targeting the elderly population, and diabetes mellitus is one of them. Thyroid dysfunction [TD] is another disease that is known to be prevalent in the elderly population. In spite of evidence of a high prevalence of TD among patients with T2D globally, there is limited information on the prevalence of TD in elderly Chinese patients with T2D. Moreover, the profile of TD prevalence among Chinese elderly patients based on clinical and demographic parameters is unknown, which might be helpful in introducing relevant guidelines. |
| Objectives | 3 | State specific objectives, including any prespecified hypotheses | 6&7 | In spite of evidence of a high prevalence of TD among patients with T2D globally, there is limited information on the prevalence of TD in elderly Chinese patients with T2D. Moreover, the profile of TD prevalence among Chinese elderly patients based on clinical and demographic parameters is unknown, which might be helpful in introducing relevant guidelines. Therefore, this cross-sectional study evaluated the prevalence of TD among elderly patients with T2D who visited endocrinology clinics in China [CROSS-DT Study]. In addition, we determined the profile of TD among enrolled patients according to the TD subtype, history of TD, gender, age, attainment of treatment goals, complications and comorbidities, and diagnosis rates. |
| Methods | | | |  |
| Study design | 4 | Present key elements of study design early in the paper | 7 | Observational cross-sectional study |
| Setting | 5 | Describe the setting, locations, and relevant dates, including periods of recruitment, exposure, follow-up, and data collection | 7 | This was an observational cross-sectional study carried out by the Chinese Association of Geriatric Research [CAGR] as a questionnaire-based survey in 24 outpatient endocrinology clinics in China between December 2015 and July 2016. The 24 study sites selected were members of CAGR and were willing to participate in the study. The study sites were spread over the first, second, third, fourth, and fifth line cities in all geographical regions of China, had tier 1, 2, and 3 hospitals, and represented different levels of economic development, ensuring that the results of the study were not biased. The leading study site was Peking University People’s Hospital with Dr Linong being the principal investigator. |
| Participants | 6 | (*a*) *Cohort study*—Give the eligibility criteria, and the sources and methods of selection of participants. Describe methods of follow-up  *Case-control study*—Give the eligibility criteria, and the sources and methods of case ascertainment and control selection. Give the rationale for the choice of cases and controls  *Cross-sectional study*—Give the eligibility criteria, and the sources and methods of selection of participants | 8 | Patients aged ≥60 years with a confirmed diagnosis of T2D as per the criteria of World Health Organization, 1999 [22] , were included in the study. Patients diagnosed with type 1 diabetes and those not willing to participate in the study were excluded from the study. Only patients who provided written informed consent were included in the study.  Data were collected using case report forms, which the sub-investigators at each clinic completed by consulting the patients and returned to the principal site. Demographic details including age, gender, race, duration for which the patients were affected by diabetes, education, history of smoking or alcoholism, and physical activities were obtained. All eligible patients also underwent a general physical examination and a medical chart review at the time of recruitment into the study. |
|  |  | (*b*) *Cohort study*—For matched studies, give matching criteria and number of exposed and unexposed  *Case-control study*—For matched studies, give matching criteria and the number of controls per case |  |  |
| Variables | 7 | Clearly define all outcomes, exposures, predictors, potential confounders, and effect modifiers. Give diagnostic criteria, if applicable | 9 | The primary end point of this study was the prevalence of TD in elderly Chinese patients with T2D. The secondary end points were ascertaining the prevalence rates by TD subtype, gender, age, and history of TD, the percentage of patients reaching treatment goals for both T2D and TD, and the percentage of complications and comorbidities in elderly patients with T2D. The diagnosis rates [proportion of patients with T2D and with previously diagnosed TD] were calculated for each TD subtype. |
| Data sources/ measurement | 8* | For each variable of interest, give sources of data and details of methods of assessment (measurement). Describe comparability of assessment methods if there is more than one group | 8 | A detailed medical history of diabetes and its complications [diabetic nephropathy, retinopathy, neuropathy, foot, and frequency of hypoglycemia], TD, hypertension, dyslipidemia, coronary heart disease, cerebrovascular disease, and other diseases was recorded. The specific treatment details regarding previous [last 6 months] and concomitant medications were also obtained. Thyroid test results [TSH, free triiodothyronine [FT3], free thyroxine [FT4], total T3, and total T4, of which TSH, FT3, and FT4 were mandatory] and other laboratory test results in the previous year were recorded, and thyroid tests were conducted for those who did not undergo thyroid tests within the past one year. Moreover, the past one year [if available] data on hemoglobin A1c [HbA1c], total cholesterol, low density lipoproteins [LDLs], triglycerides, electrocardiograms, and bone density were collected. Missing data for demographic and laboratory parameters were recorded in the case report form. |
| Bias | 9 | Describe any efforts to address potential sources of bias |  | NA |
| Study size | 10 | Explain how the study size was arrived at | 9 | In order to detect an expected prevalence rate of 15%, with 2% precision and 95% confidence, 1650 subjects were required. The sample size was calculated as follows:  n = Z^2^p[1-p]/d^2^ |

Continued on next page

| Quantitative variables | 11 | Explain how quantitative variables were handled in the analyses. If applicable, describe which groupings were chosen and why | 10 | | For continuous variables [age, height, and body mass index], the results were summarized using the number of observations [N], number of missing data points [miss], means, standard deviations [SDs], medians, 25th percentiles [Q1], 75th percentiles [Q3], minimums, and maximums. For categorical variables [gender and hypertension], the results were summarized as frequencies and percentages. |
| --- | --- | --- | --- | --- | --- |
| Statistical methods | 12 | (*a*) Describe all statistical methods, including those used to control for confounding | 10 | Student’s t-test and chi-square test/Fisher’s exact test were used to determine the homogeneity of baseline characteristics. All statistical tests were two-sided and p<.05 was considered significant.  The statistical analysis was carried out using statistical analysis system [SAS] version 9.4 [SAS Institute Inc., Cary, North Carolina, USA]. | |
|  |  | (*b*) Describe any methods used to examine subgroups and interactions |  | NA | |
|  |  | (*c*) Explain how missing data were addressed |  | NA | |
|  |  | (*d*) *Cohort study*—If applicable, explain how loss to follow-up was addressed  *Case-control study*—If applicable, explain how matching of cases and controls was addressed  *Cross-sectional study*—If applicable, describe analytical methods taking account of sampling strategy | 9 | In order to detect an expected prevalence rate of 15%, with 2% precision and 95% confidence, 1650 subjects were required. The sample size was calculated as follows:  n = Z^2^p[1-p]/d^2^ | |
|  |  | (*e*) Describe any sensitivity analyses |  | NA | |
| Results | | | | | |
| Participants | 13* | (a) Report numbers of individuals at each stage of study—eg numbers potentially eligible, examined for eligibility, confirmed eligible, included in the study, completing follow-up, and analysed | 10 | We enrolled 1677 patients [mean ± SD age: 71.17 ± 8.06 years; 882 [52.59%] male and 795 [47.41%] female patients] from 24 endocrinology centers across China. The demographic and baseline characteristics of the patients are summarized in Table 1. Figure 1, describes the patient enrolment according to eligibility criteria. | |
|  |  | (b) Give reasons for non-participation at each stage |  | NA | |
|  |  | (c) Consider use of a flow diagram |  | Figure 1 | |
| Descriptive data | 14* | (a) Give characteristics of study participants (eg demographic, clinical, social) and information on exposures and potential confounders | 10-14 | Table 1 &2 | |
|  |  | (b) Indicate number of participants with missing data for each variable of interest |  | NA | |
|  |  | (c) *Cohort study*—Summarise follow-up time (eg, average and total amount) |  | NA | |
| Outcome data | 15* | *Cohort study*—Report numbers of outcome events or summary measures over time |  | NA | |
|  |  | *Case-control study—*Report numbers in each exposure category, or summary measures of exposure |  | NA | |
|  |  | *Cross-sectional study—*Report numbers of outcome events or summary measures | 10-18 | 4 | |
| Main results | 16 | (*a*) Give unadjusted estimates and, if applicable, confounder-adjusted estimates and their precision (eg, 95% confidence interval). Make clear which confounders were adjusted for and why they were included | 15-18 | 1. Prevalence TD in patients with T2D: overall and by age, gender, TD subtype and history of TD  2. Achievement of treatment goals  3. Complications and comorbidities  4. Diagnosis rate by TD subtype | |
|  |  | (*b*) Report category boundaries when continuous variables were categorized |  | NA | |
|  |  | (*c*) If relevant, consider translating estimates of relative risk into absolute risk for a meaningful time period |  | NA | |

Continued on next page

| Other analyses | 17 | Report other analyses done—eg analyses of subgroups and interactions, and sensitivity analyses |  | NA |
| --- | --- | --- | --- | --- |
| Discussion | | | | |
| Key results | 18 | Summarise key results with reference to study objectives | 18 | This study, evaluated the prevalence of TD in elderly patients with T2D in 24 endocrinology clinics across China and found that TD was underdiagnosed in this population. Our findings suggest that regular annual screening for thyroid function in patients with T2D will help improve health outcomes and quality of life in elderly patients with T2D |
| Limitations | 19 | Discuss limitations of the study, taking into account sources of potential bias or imprecision. Discuss both direction and magnitude of any potential bias | 21 | One of the limitations of this study is its cross-sectional design. The sample was derived from an outpatient setting and may not be representative of the true population. Also, there is a possibility of selection bias because the patients were already under medical care. Although 24 endocrinology centers were in iodine sufficient areas, we did not determine urinary iodine concentrations. In addition, our study did not evaluate the impact of diabetes-related risk factors on TD, though, a previous study revealed no significant relationships between and diabetes-related clinical parameters such as duration of diabetes, HbA1c levels and diabetic complications with TD. |
| Interpretation | 20 | Give a cautious overall interpretation of results considering objectives, limitations, multiplicity of analyses, results from similar studies, and other relevant evidence | 22 | TD is prevalent in Chinese patients with T2D, with clinical- hypothyroidism being the most common TD subtype, which was more prevalent in women than in men. In addition, dyslipidemia, osteoporosis, and CHD are common complications in elderly Chinese patients with both T2D and TD. We recommend routine thyroid function testing of elderly patients with T2D in outpatient settings, especially of elderly females, and other old patients with risk factors, including those with a family history of diabetes, TD, and cardiovascular disease. |
| Generalisability | 21 | Discuss the generalisability (external validity) of the study results | 20 | In our study, the most common complications and comorbidities in T2D were diabetic neuropathy [43.46%], cataracts [24.73%], and diabetic retinopathy [22.68%]. In past, the prevalence of diabetic complications has been assessed in several studies in patients with comorbid TD. According to some studies, subclinical hypothyroidism is an independent risk factor for severe diabetic retinopathy in patients with T2D. However, other studies report controversial risks of diabetic retinopathy in patients with TD and T2D. Altogether, these previous reports and our present findings support early screening for TD during the course of the treatment for T2D. |
| Other information | |  | | |
| Funding | 22 | Give the source of funding and the role of the funders for the present study and, if applicable, for the original study on which the present article is based | 22 | The study was funded by Merck Serono China Co. Ltd., an affiliate of Merck KGaA, Darmstadt, Germany. |
| S1 Checklist |  | S1 Fig  S2 Fig  S1 Table  S2 Table  S3 Table |  | Flow chart showing the distribution of the population with thyroid dysfunction [TD] among elderly Chinese patients with type 2 diabetes [T2D].  Prevalence of diabetic complications in elderly Chinese patients with type 2 diabetes.  Demographic and baseline characteristics of elderly, T2D patients with and without previously diagnosed TD.  Baseline medical history and course of disease in elderly, T2D patients.  Patient profiles, including comorbidities and family history, for elderly T2D patients suffering with and without previously diagnosed TD. |

*Give information separately for cases and controls in case-control studies and, if applicable, for exposed and unexposed groups in cohort and cross-sectional studies.

**Note:** An Explanation and Elaboration article discusses each checklist item and gives methodological background and published examples of transparent reporting. The STROBE checklist is best used in conjunction with this article (freely available on the Web sites of PLoS Medicine at http://www.plosmedicine.org/, Annals of Internal Medicine at http://www.annals.org/, and Epidemiology at http://www.epidem.com/). Information on the STROBE Initiative is available at www.strobe-statement.org.
